# Accurate estimation of concrete consumption in tunnel lining using terrestrial laser scanning

**DOI:** 10.1038/s41598-023-51132-x

**Published:** 2024-02-01

**Authors:** Liao Jian, Wenge Qiu, Yunjian Cheng

**Affiliations:** 1 School of Civil Engineering, Key Laboratory of Transportation Tunnel Engineering, Ministry of Education, Southwest Jiaotong University, No. 111, North Section, Second Ring Road, Jinniu District, Chengdu, 610031 Sichuan China; 2 Chengdu Tianyou Tunnelkey Co., Ltd., Chengdu, 610031 Sichuan China; 3 School of Civil Engineering and Geomatics, Southwest Petroleum University, Chengdu, 610500 Sichuan China

**Keywords:** Civil engineering, Lasers, LEDs and light sources

## Abstract

Accurate estimation of concrete (including shotcrete) consumption plays a crucial role in tunnel construction. A novel method has been introduced to accurately estimate concrete consumption with terrestrial laser scanning (TLS). The estimation needs to capture TLS data of tunnel surfaces at different stages of construction. Unrolling point clouds, a novel two-stage algorithm consisting of noise removal and hole filling has been used to generate resampled points. Furthermore, resampled points from two scans (before and after lining construction) ultimately generate an innovative computation model composed of multiple hexahedral elements, which is used for calculating volumes. The proposed technique was applied to the Tiantaishan highway tunnel and Da Fang Shan high-speed railway tunnel. The calculation relative error of the rebound rate is 0.19%, and the average relative error in predicting the demand for secondary lining concrete is 0.15%. Compared with 3D Delaunay with curve fitting, the proposed technique offers a more straightforward operation and higher accuracy. Considering factors such as tunnel geometry, support design, and concrete properties, a computational model will provide valuable insights into optimizing resource allocation and reducing material waste during construction.

## Introduction

Tunnels are engineering structures buried within geological layers, representing a crucial form of utilizing underground spaces for human activities. Tunnels can be categorized into various types, such as road tunnels, railway tunnels, water supply tunnels, municipal tunnels, and mining tunnels. The selection of an appropriate tunnel excavation method is vital for tunnel construction, improving construction efficiency, reducing engineering costs, and simultaneously ensuring construction safety^1,2^. With the development of China’s transportation infrastructure, a large number of new tunnels are constructed every year. Conventional tunnelling is widely applied in tunnel construction due to its broad geological applicability, minimal disturbance to surrounding rocks, and the versatility of support forms it offers. This excavation method utilizes millisecond and smooth blasting techniques to excavate the entire face, with composite primary and secondary linings formed to construct the tunnel structure^3^. Tunnel lining is a permanent structure that supports and maintains the long-term stability and durability of a tunnel. Its functions include providing support, maintaining the stability of the tunnel, preserving the space required for train operation, preventing weathering of the surrounding rock, and mitigating the impact of groundwater^4^. Primary linings are formed by directly spraying wet mix shotcrete on excavated rock surfaces. Shotcrete plays a significant role during tunnel construction as it provides structural support. Secondary linings refer to the reinforced concrete or plain concrete lining constructed on the inner side of primary linings during tunnel construction. Primary linings and secondary linings form composite linings. Primary linings aim to maximize the bearing capacity of the surrounding rock, while secondary linings serve as safety reserves and decorative coverings. In the process of constructing tunnels, a large amount of concrete is needed for primary and secondary linings. Therefore, accurate estimation of concrete consumption is crucial for tunnel construction^5^, as it directly influences cost management, resource allocation, and project efficiency. By calculating the shotcrete volume sprayed on excavated rock surfaces, we can not only determine the primary lining volume but also calculate the rebound rate of the shotcrete^6,7^. Rebound rate regarded as a crucial indicator for evaluating the performance of shotcrete, has always been highly valued by researchers in the field of construction materials^8–10^. The rebound rate of shotcrete during the construction of primary linings is used to estimate the amount of shotcrete prepared during the construction process^11^. In this way, materials can be saved to achieve cost-effectiveness. Predicting the demand for secondary lining concrete also holds positive significance in saving concrete preparation. Therefore, it is necessary to estimate concrete consumption during tunnel construction.

Traditionally, measurers measure several points on tunnel surfaces with a total station to calculate the weighted average overbreak and underbreak values^12^. Concrete consumption is then estimated by multiplying this value by the perimeter of the designed sprayed area. This method has low data acquisition efficiency, and the accuracy of estimation relies on the experience of the measurers. To enhance acquisition efficiency, terrestrial laser scanning (TLS) is used to capture detailed information about a tunnel structure. TLS utilizes high-resolution laser scanners to capture detailed point cloud data sets of tunnel surfaces^13–15^. TLS has been extensively utilized for non-destructive testing during both construction and operation^16,17^. By analyzing the geometric shape under construction, it is possible to quickly assess the excavation quality and deformation^18–22^. In addition, conducting two scans with TLS after tunnel excavation and after lining construction allows for a thickness model of tunnel lining, ensuring structural safety^23^. On this basis, a finite element model based on an actual tunnel can be established using points obtained from multiple scans at different construction stages^24^. Due to the characteristics of high speed, high precision, and large data volume of TLS, we also employ TLS to acquire tunnel points. Based on this data, concrete consumption is calculated.

To the best of our knowledge, volume calculations with TLS have been studied in many fields^25–29^. Brede et al.^30^ utilized the TreeQSM method for tree modelling and trunk volume calculations with novel unmanned aerial vehicle laser scanning (UAV-LS). Singh et al.^31^ proposed an approach for tree volume estimation using RANSAC and RHT algorithms from TLS data sets. Through quantitative structure modelling, the calculation method of tree volume with TLS is more suitable for biomass assessment^32,33^. Xu et al.^14^ generated digital elevation models (DEMs) for open-pit mines and then calculated the exploitative volume through DEM differentials. Clearly, this method, due to its simplicity in calculation, is more suitable for large-scale irregular object volume calculations. However, in construction, there is still limited research on estimating concrete consumption through volume calculations of points. Slattery et al.^34^ established a triangulated irregular network (TIN) with TLS to determine earthwork quantities. Although 3D Delaunay is commonly used for calculating irregular volumes, it often performs poorly when points have a high level of noise, numerous digital holes, and low density. Hu et al.^35^ sliced the point clouds before and after excavation. They then integrate the sliced areas in the thickness direction to compute the excavation volume. However, this method is evidently not suitable for calculating the object volumes with irregular surfaces. Huang et al.^12^ proposed using a photogrammetric system to reconstruct a tunnel point cloud model. They calculate overbreak and underbreak volumes by multiplying deviation areas by average linear deviations. Due to the inconsistency in deviation areas of the tunnel lining's inner and outer surfaces, this approximate calculation method yields large relative errors, especially in the case of curved tunnel sections. Therefore, there is a need for a new, efficient, and high-precision point cloud volume calculation method for a tunnel in construction. Results obtained through volume calculation can then be used to assess concrete consumption.

Therefore, the main objective of this study is to establish a concrete consumption calculation model for tunnel construction with TLS. This method primarily involves innovations in two aspects: (i) noise reduction and hole filling based on tunnel design data and geometric features, and (ii) a novel approach to establishing a concrete consumption calculation model for a tunnel in construction. Modelling results are used to automatically create tunnel concrete consumption and thickness models. Additionally, the algorithm developed in this study can be applied to both straight and curved tunnels, and its adaptability has been validated using TLS data from two tunnels.

## Related works

There are three key techniques for estimating concrete consumption using TLS: establishment of a volume calculation model, noise reduction, and filling digital holes.

### Establishment of a volume calculation model

So far, a significant number of scholars have studied tunnel engineering modelling. Sharafat et al.^36^ proposed a novel BIM-based multimodel tunnel information modelling (TIM) framework. Duan et al.^37^ established a practical shield tunnel segment model based on point cloud data, which can effectively assess the misalignment of shield tunnel segments. To enhance management efficiency and economic benefits in tunnel construction, volume calculation is widely employed, including but not limited to volume calculation in earthwork and concrete applications. Tanoli et al.^38^ created a 3D earthwork section design based on a parametric modelling technique. Through the earthwork design sections, the volume of earthwork can be effectively estimated. A crucial aspect of calculating concrete consumption with TLS lies in the volume calculation of points. Currently, there are various methods for point cloud volume calculation^28,39^. Lee and Lee^40^ calculated earthwork quantities with 3D Delaunay to establish a polyhedral model. However, it is obvious that 3D Delaunay may overlook the geometric features when point cloud density is low^41–43^. In another study, Li et al.^44^ calculated point cloud volumes by slicing point clouds, integrating the area enclosed by point clouds on each slice along the perpendicular direction to the cutting direction, and then summing these results. The thinner the slicing thickness in this method, the higher the accuracy, but the lower the computational efficiency. Additionally, this approach is sensitive to the noise points and digital holes in point clouds^45^. To mitigate the adverse impact of digital holes, Shah et al.^46^ employed the 3D convex hull method to estimate the volume of raw materials inside the silo. However, this method exhibits significant volume calculation errors for non-convex models. Han et al.^47^ calculated the volume and biomass of large trees with 3D alpha shape. This method provides more accurate volume calculations for concave models. Besides, Xu et al.^14^ first applied 3D Delaunay to point clouds. After projecting it onto the $X - Y$ plane, they connected the projected triangular meshes with the original triangular meshes to form multiple tetrahedra. Calculating the volumes of these tetrahedra provided the volume between point clouds and the $X - Y$ plane. The volume difference between the two scans then yielded the exploitative volume over the open-pit mine. This method performs well in calculating stack volumes. It's worth noting that volume calculations of a large number of points require substantial computational effort. So, Liu et al.^48^ accelerated the efficiency of point cloud volume calculation through graphics processing unit (GPU) rendering technology. Xu et al.^49^ proposed an efficient 3d volumetric object representation (VOLA) that enabled significant savings in memory usage for volumetric data. Tommy et al.^50^ voxelized points to produce a model made up of voxels, and then calculated the volume of the voxel model. The accuracy and efficiency depend on the voxel size^51^.

Of course, for certain specific computational scenarios, a more accurate calculation model can be established by combining several points with their geometric features. These approaches aim to achieve more precise volume calculations. Knyva et al.^25^ measured points on the surfaces of an oil tank with a total station. Based on the geometric features of the oil tank, they established a geometric model and performed volume calculations. Liu et al.^48^ calculated the angles and base areas information of the coal pile from the collected points, thus establishing the geometric model of these coal piles. Volume calculation methods for these regular models are based on specific scenarios, making it challenging to directly apply them to concrete consumption in tunnel engineering. As tunnel construction involves referencing a series of standards and design data, it is not entirely irregular. Therefore, it is evidently necessary to establish a concrete consumption calculation model for tunnel engineering based on design data and geometric features constrained by standards.

### Noise reduction

During tunnel construction, raw points collected with TLS are often a set of unorganized points. To enhance the value of raw points, it is necessary to filter out subsets logically belonging to the tunnel structure while removing the subsets formed by non-tunnel structures, such as workers and cables. These non-tunnel structure points are considered noise in the process of establishing a concrete consumption calculation model. Since noise points are often not on the surfaces of the object to be calculated, noise points significantly impact volume calculations. Methods directly applied to denoise typically rely on local normal, principal curvature, or geometric features. For points of regular objects, achieving high-precision denoising is relatively straightforward. Cheng et al.^52^ divided the point clouds of an operational railway tunnel into lining and different components based on relative distance, relative angle, convex hull, and linearity. In another study, Duan et al.^37^ proposed the use of shield tunnel depth unfolding diagrams and cylinder fitting to remove outliers. This method achieves excellent denoising results for the shield tunnel points. However, it is challenging to apply this approach to the inner surfaces of primary linings. Complex denoising methods evidently reduce the efficiency of point cloud denoising. Du et al.^53^ removed shield tunnel appendages quickly by unfolding tunnel point clouds and adjusting the parameters of the cloth simulation filtering algorithm. Shen et al.^54^ employed two control points to remove non-lining points from point clouds of an operational shield tunnel. However, for tunnels under construction, obtaining two control points on irregular tunnel surfaces can be challenging. For points on irregular surfaces, directly denoising through geometric features makes it difficult to achieve satisfactory results. Often, denoising involves segmenting point clouds by calculating the variation in normal vectors, thereby combining geometric features for denoising. Zhang et al.^55^ provided feature-preserved point cloud simplification (FPPS) for point cloud simplification. Point cloud simplification with preserving the data features can partially reduce noise, but it also significantly reduces the density of point clouds. Ankang Ji et al.^56^ proposed a deep learning method named semi-supervised learning-based point cloud network (SPCNet) to boost segmentation by alleviating labelling tasks in tunnel engineering. It requires a significant amount of manual involvement to improve the accuracy of segmentation.

### Filling digital holes

In addition to noise points, point clouds often exhibit digital holes due to missing data, primarily resulting from limitations in data collection techniques and complex structures. Digital holes are often detrimental to volume calculation. Large digital holes in point clouds tend to reduce the point cloud density and significantly impact the geometric characteristics. Therefore, appropriately filling digital holes is crucial for accurate volume calculation. Quinsat and Lartigue^57^ filled digital holes based on the prior knowledge of the numerical model as a nominal mesh. Li et al.^58^ connected multi-source data sets accurately with the Laplace differential domain fusion approach. To solve the time-consuming issue of filling digital holes in multi-source data scans of the same object from different angles, Middleton et al.^59^ filled the areas of low point density (or small surface holes) with large voxels. However, voxels yield limited effectiveness for large digital voids. In this context, Hu et al.^60^ formulated the hole-filling step as an optimization problem based on the selected most similar area and regularized by the graph-signal smoothness prior.

## Methodology

**Figure 1:**
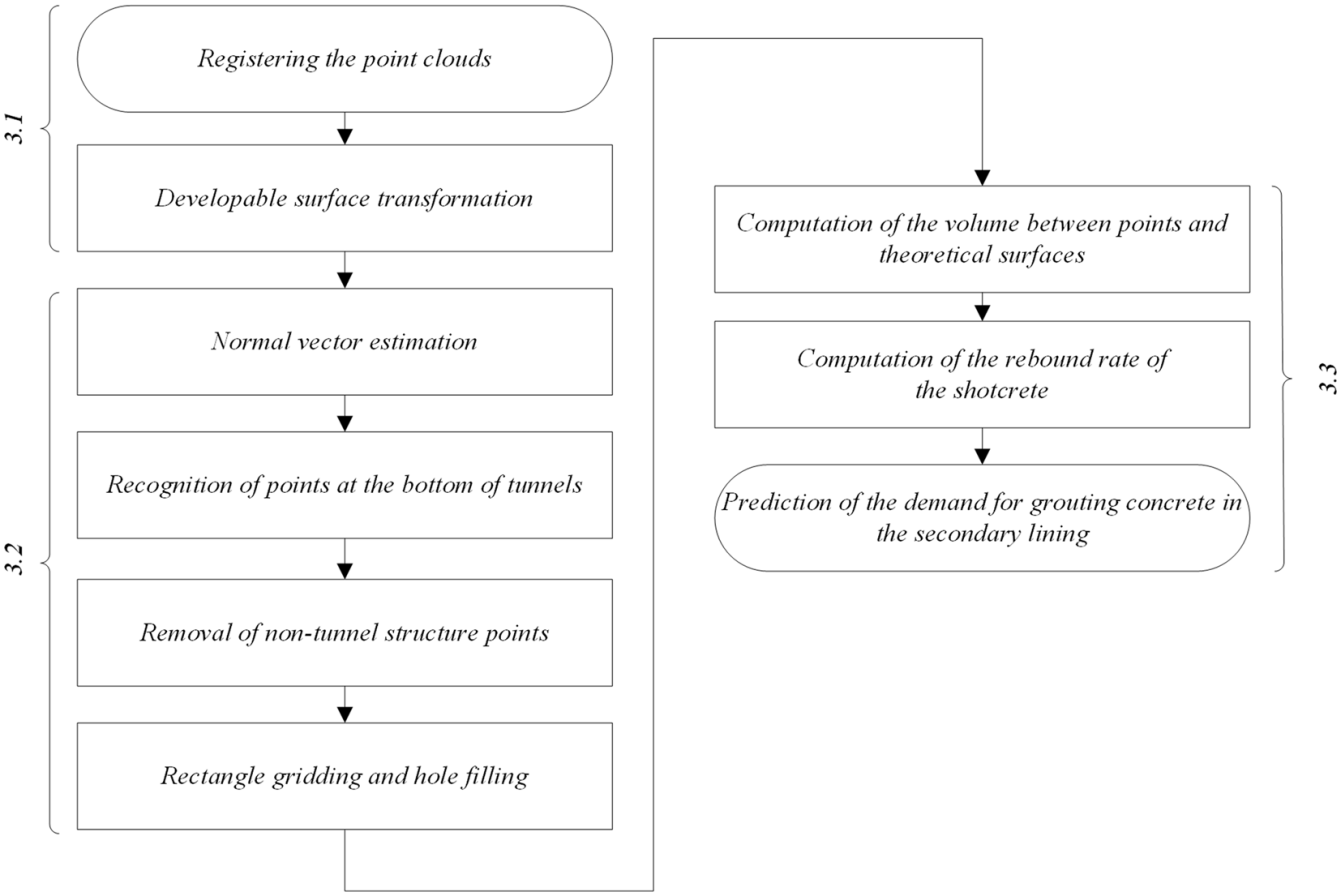
Flowchart of the proposed technique.

Conventional tunnelling is widely adopted in tunnel engineering for its ability to rely on the bearing capacity of the surrounding rock. This excavation method utilizes millisecond and smooth blasting techniques to excavate the entire face, with composite primary and secondary linings formed to construct the tunnel structure. Primary linings consist of shotcrete, anchor bolts, steel mesh, steel support structures, and other materials. It must be installed immediately after tunnel excavation has taken place to maintain stability through the cavity effect. During the process, the redistribution of stress in the surrounding rock facilitates adjusting to achieve a new balance between primary linings and the surrounding rock, maximizing the inherent strength of the surrounding rock and making use of its self-supporting capability^3^. Surrounding rock is primarily responsible for the main burden of stress. Therefore, primary linings aim to maximize the bearing capacity of the surrounding rock, while secondary linings serve as a safety reserve and decorative covering. Therefore, to estimate concrete consumption in tunnel engineering, it is necessary to scan the tunnel before and after concrete construction. As shown in Fig. 1, this study utilized TLS to collect raw point cloud data of a tunnel, and established a concrete consumption calculation model using the raw data in the following sequence:

1. Pretreatment of point clouds. Including registering point clouds and developable surface transformation;
2. Noise reduction and hole filling according to design data and construction standards;
3. Establishment of the concrete consumption model.

### Pretreatment of point clouds

#### Registering the point clouds

Raw tunnel point clouds are obtained using TLS. Since the scanner saves data based on its local coordinate system during scanning, the local coordinate systems of this scanner differ between any two scans. To ensure that point clouds directly reflect their actual positions, coordinate registration is required. The transformation between two different coordinate systems involves rotation and translation, and at least three common points in two coordinate systems are needed to solve them. Fortunately, TLS is equipped with an inclinometer, which ensures that the vertical direction of TLS aligns with the geodetic coordinate system. Therefore, rotation and translation can be solved with just two common points. During scanning with TLS, two spherical prisms provided are chosen as common points. By using a total station and control points in the geodetic coordinate system, the coordinates of spherical prisms are obtained in the geodetic coordinate system. By identifying the spherical prisms on the unfolded map and performing sphere fitting, the coordinates of the spherical prisms are obtained in the local coordinate system^52^. Point clouds are registered in the geodetic coordinate system using two common points and a vertical constraint condition. To eliminate cumulative errors generated by direct concatenation, each scan has been registered individually. After that, the tunnel points are combined with tunnel design data. The density of points obtained by TLS significantly reduces as the scanner gets farther from objects. Therefore, multi-locations can ensure the density of point clouds.

#### Developable surface transformation

**Figure 2:**
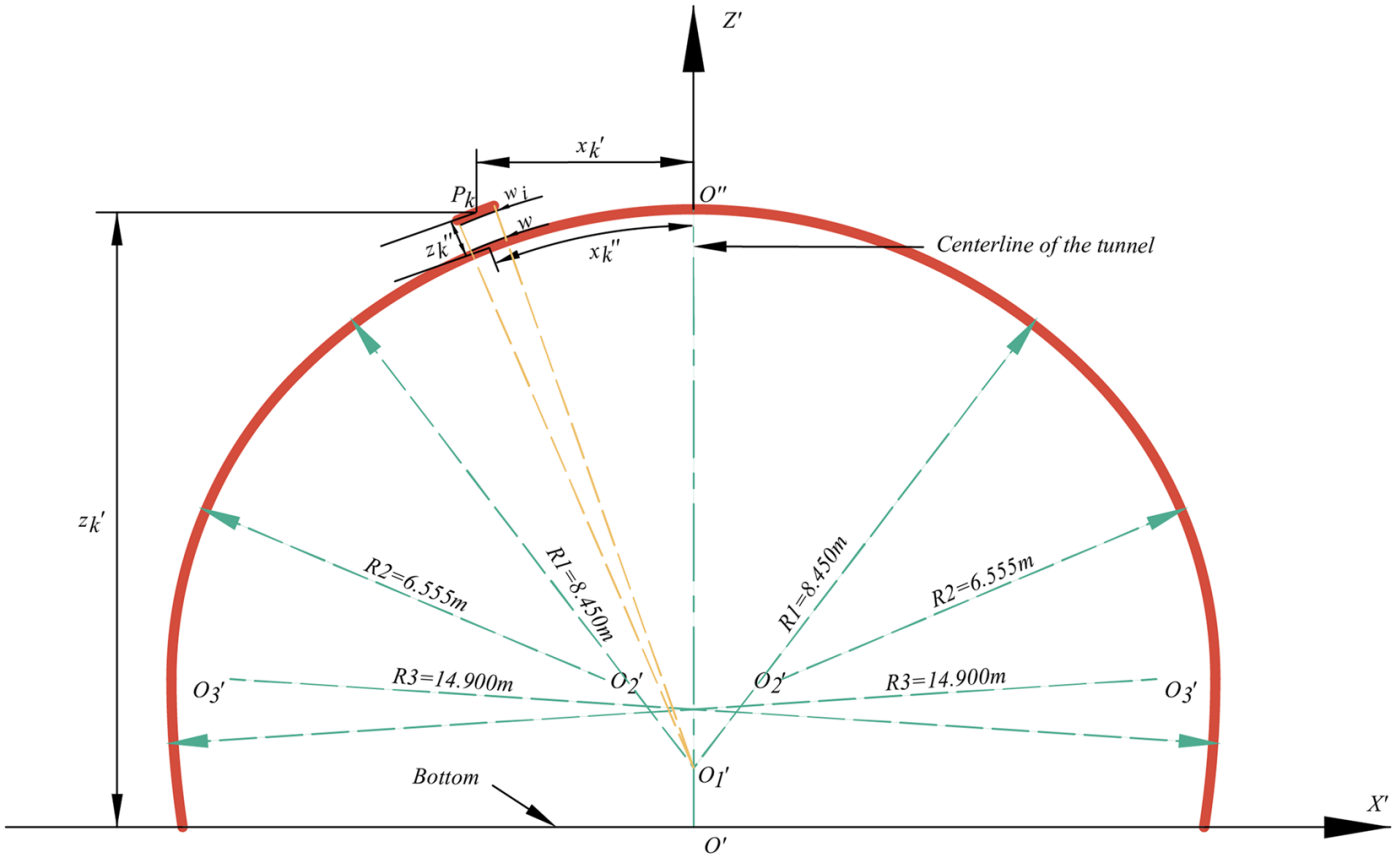
Theoretical cross-section at the starting station mileage.

**Figure 3:**
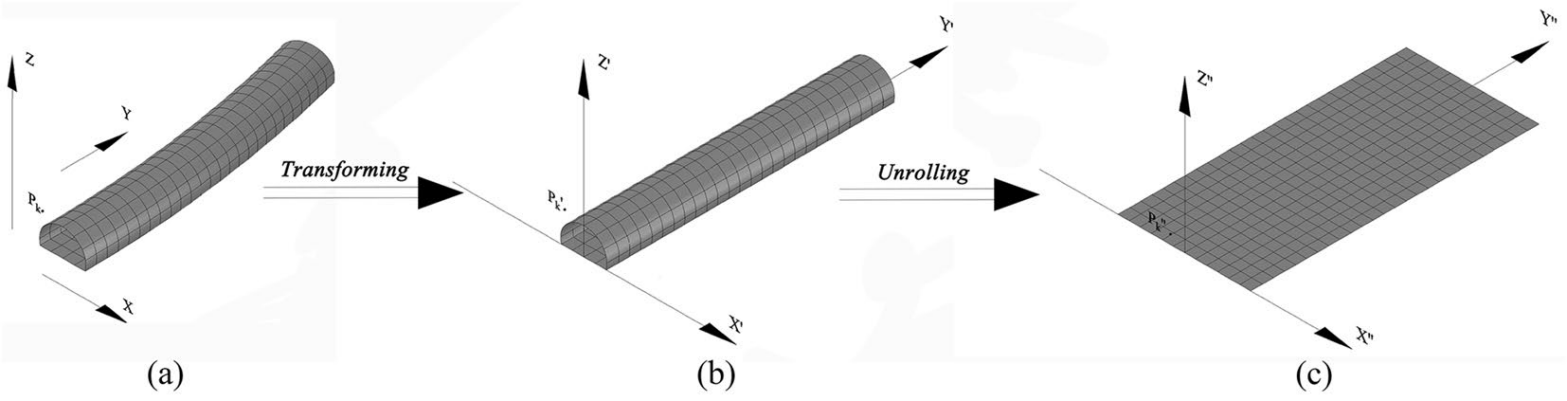
Reshaping a curved tunnel surface into a developable surface: (**a**) geodetic coordinate system; (**b**) tunnel stationing coordinate system; (**c**) unrolling.

Unwrapped point clouds should be generated following the method proposed by the authors^61^. The main characteristic of this method is to transform an undevelopable surface of tunnel curve segments into a developable surface. The registered point cloud obtained by TLS includes cartesian coordinate values and intensity values. Figures 2 and 3 show a registered element $P_{k}$ after coordinate registration. The grey surface model is the theoretical inner contour of linings known as the theoretical surfaces in Fig. 3a. A new coordinate system shown in Fig. 3b is called tunnel stationing coordinate system (TSCS) because of its position with respect to the tunnel’s stations^61^. Theoretical surfaces and point $P_{k}$ are transformed from the geodetic coordinate system into TSCS. The station axis $Y^{\prime }$ is a straight line generated from a length-preserving transformation of the design centerline in the geodetic coordinate system. Then, developable surfaces are cut along the cross-sectional line which intersects the negative $Y^{\prime }$ axis and is unrolled into a flat plane, as demonstrated in Fig. 3c.

### Noise reduction and hole filling

**Figure 4:**
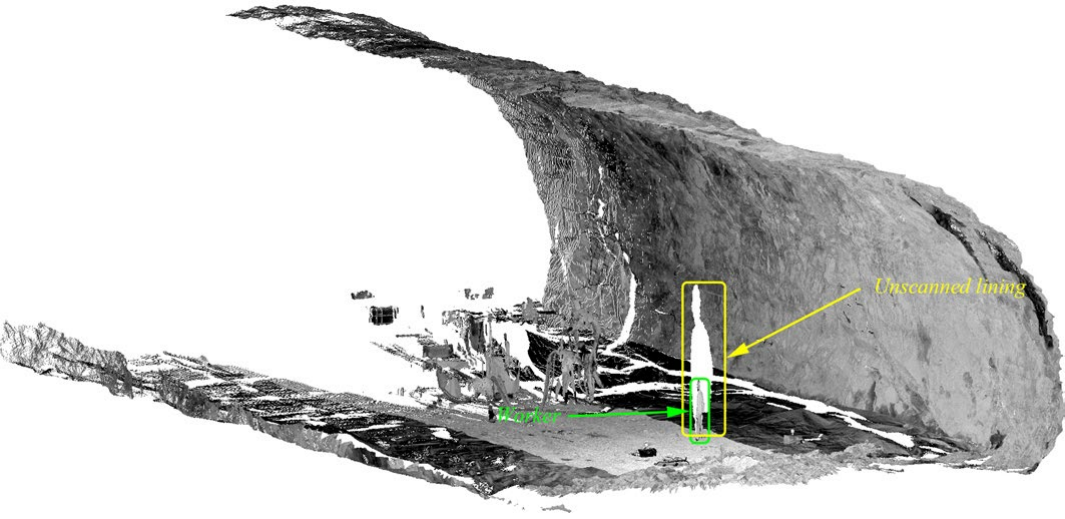
Holes and noise points of a raw point cloud.

While scanning a tunnel, TLS may scan obstacles near tunnel surfaces, such as workers and pipelines, as shown in Fig. 4. To ensure accuracy, it is necessary to remove non-tunnel structure points. However, there are still unscanned areas behind the obstacles shown in Fig. 4, which appear as holes in this point cloud. Additionally, uneven linings have caused other holes in the dataset, which can adversely affect the calculation accuracy.

Therefore, this paper proposes a two-stage algorithm for generating points of concrete consumption, consisting of noise reduction and hole filling. In the initial stage, remove significant non-tunnel structure points based on the local normal vector changes. In the final stage, holes are filled with gridding bilinear interpolation/extrapolation techniques in unrolling point clouds.

#### Normal vector estimation

The effectiveness of point cloud denoising through calculating the variation in point normal vectors has been confirmed by multiple studies^37,56,62^. Therefore, we first calculate the normal vectors of points based on the principal component analysis (PCA)^63^. Suppose $N_{i}$ is a point in TSCS and 20 k-nearest neighbours of $N_{i}$ are composed of $K_{j} (j = 1,2, \ldots ,20)$. To calculate a raw normal vector $\vec{n}_{i}$ of $N_{i}$, the objective function is:

1 $$\mathop {\min }\limits_{{\left| {\vec{n}_{i} = 1} \right|}} \sum\limits_{j = 1}^{20} {((K_{j} - C)^{T} \cdot \vec{n}_{i} )^{2} }$$

where $C = \frac{1}{20}\sum\limits_{j = 1}^{20} {K_{j} }$ is the geometric centre of $K_{j} (j = 1,2,...,20)$.

**Figure 5:**
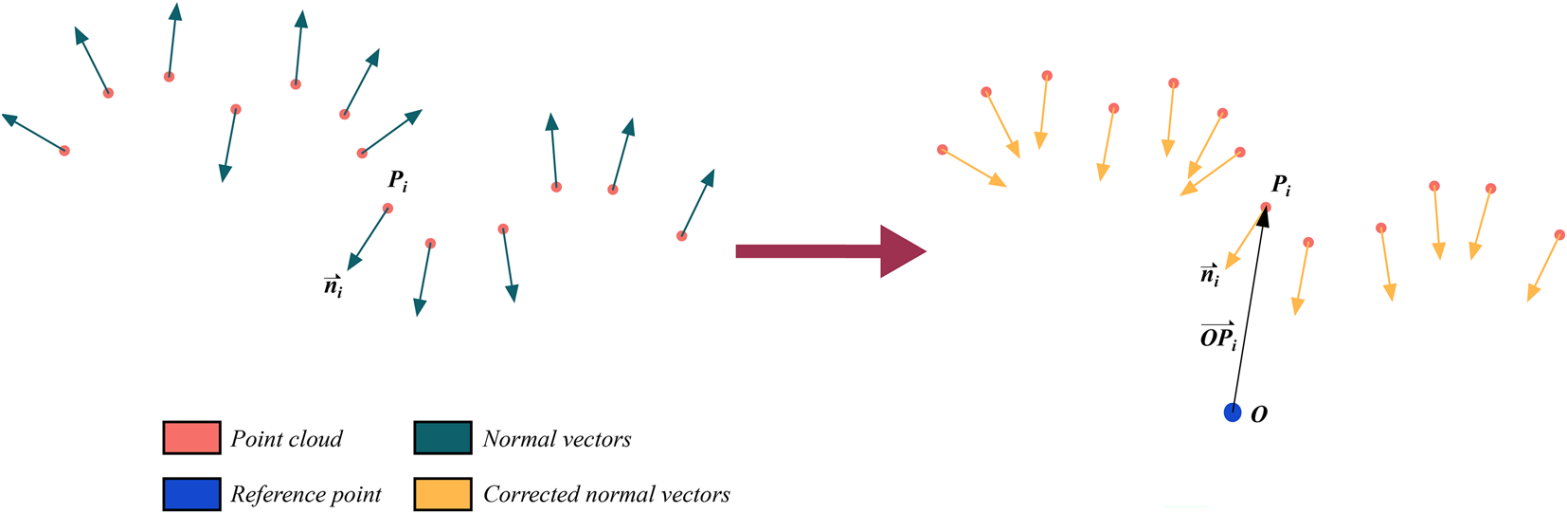
Normal vector estimation.

As illustrated in Fig. 5, these raw normal vectors are located both outside and inside tunnel surfaces. To calculate the true variation in normal vectors between adjacent points, we need to adjust normal vectors to face the interior of the tunnel. In a single scan, we have selected a reference point $O(x_{0} ,y_{0} ,z_{0} )$, which is transformed from the origin $(0,0,0)$ in the local coordinate system. Together, these points $N_{i} (x_{i} ,y_{i} ,z_{i} )(i = 1,2, \ldots ,n)$ and the reference point $O(x_{0} ,y_{0} ,z_{0} )$ form new vectors $\overrightarrow {{ON_{i} }} (i = 1,2,...,n)$. We then measure the angles $\alpha_{i} (i = 1,2,...,n)$ between $\overrightarrow {{ON_{i} }} (i = 1,2,...,n)$ and $\vec{n}_{i} (i = 1,2,...,n)$ as follows:

2 $$\alpha_{i} = \left| {\arccos [{{(\overrightarrow {{OP_{i} }} \cdot \vec{n}_{i} )} \mathord{\left/ {\vphantom {{(\overrightarrow {{OP_{i} }} \cdot \vec{n}_{i} )} {(\left| {\overrightarrow {{OP_{i} }} } \right| \cdot \left| {\vec{n}_{i} } \right|)}}} \right. \kern-0pt} {(\left| {\overrightarrow {{OP_{i} }} } \right| \cdot \left| {\vec{n}_{i} } \right|)}}]} \right|$$

when $\alpha_{i} > {\pi \mathord{\left/ {\vphantom {\pi 2}} \right. \kern-0pt} 2}$, $\vec{n}_{i} = - \vec{n}_{i}$. Normal vectors $\vec{n}_{i} (i = 1,2, \ldots ,n)$ can be corrected as shown in Fig. 5.

#### Recognition of points at the bottom of tunnels

After normal vector estimation, the corresponding point set $A_{1}$ whose normal vectors have an angle less than $\frac{\pi }{2}$ with vector $(0,0,1)$ are retained. However, there are still some lining points remaining and some tunnel bottom points removed. Point set $A_{1}$ is fitted to form a plane $P_{{A_{1} }} (a_{1} x + b_{1} y + c_{1} z + d_{1} = 0)$. The points in set $A_{1}$ that have a distance less than $t_{1}^{d}$ to the plane $P_{{A_{1} }}$ form a new set $A_{2}$:

3 $$d_{i}^{\prime } = {{\left| {a_{1} x_{i} + b_{1} y_{i} + c_{1} z_{i} + d_{1} } \right|} \mathord{\left/ {\vphantom {{\left| {a_{1} x_{i} + b_{1} y_{i} + c_{1} z_{i} + d_{1} } \right|} {\sqrt {a_{1}^{2} + b_{1}^{2} + c_{1}^{2} } }}} \right. \kern-0pt} {\sqrt {a_{1}^{2} + b_{1}^{2} + c_{1}^{2} } }}$$

4 $$t_{1}^{d} = 3\sqrt {{{\sum\limits_{i = 1}^{m} {(d_{i}^{\prime } )^{2} } } \mathord{\left/ {\vphantom {{\sum\limits_{i = 1}^{m} {(d_{i}^{\prime } )^{2} } } m}} \right. \kern-0pt} m}}$$

**Figure 6:**
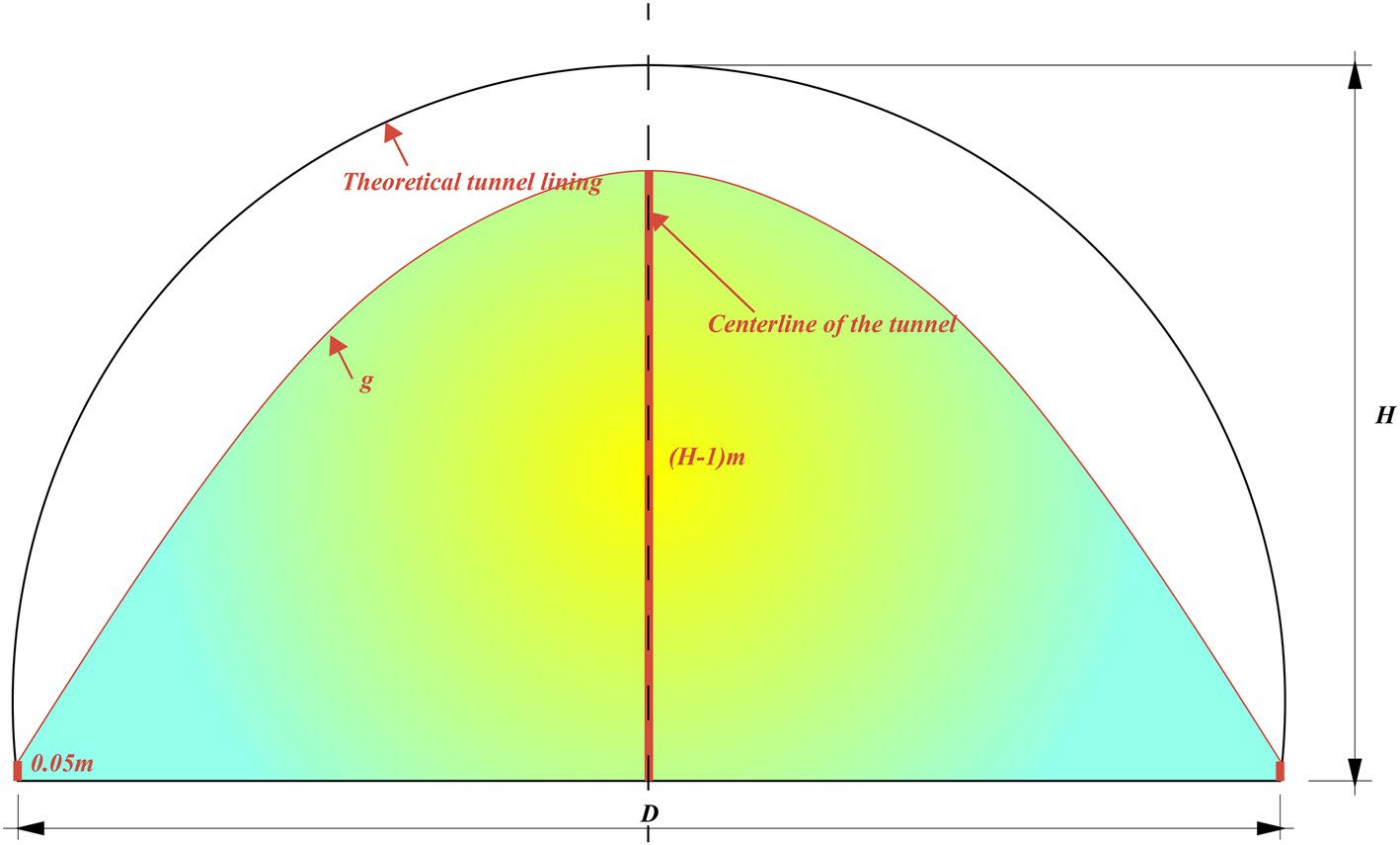
The value range of $g$.

**Figure 7:**
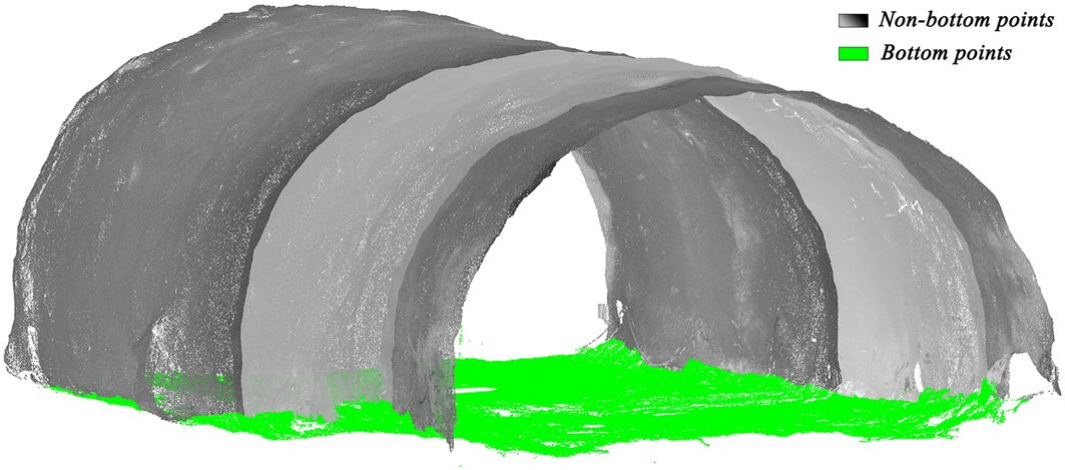
Removal of bottom points.

Fit point set $$A_{2}$$ to a plane $$P_{{A_{2} }}$$ to update $$t_{1}^{d}$$ to $$t_{2}^{d}$$, and then a new set $$A_{3}$$ is collected. Until a point set $$A_{n}$$ is obtained ($$t_{n}^{d} - t_{n - 1}^{d} < = 10^{ - 5}$$). And the best-fitting plane $$P_{{A_{n} }}$$$$(a_{n} x + b_{n} y + c_{n} z + d_{n} = 0)$$ is finally obtained. $$D_{i} (i = 1,2, \ldots ,n)$$ are the distances between points $$N_{i} (i = 1,2, \ldots ,n)$$ and the best-fitting plane $$P_{{A_{n} }}$$$$(a_{n} x + b_{n} y + c_{n} z + d_{n} = 0)$$ as follows:

5 $$D_{i} = {{\left| {a_{n} x_{i} + b_{n} y_{i} + c_{n} z_{i} + d_{n} } \right|} \mathord{\left/ {\vphantom {{\left| {a_{n} x_{i} + b_{n} y_{i} + c_{n} z_{i} + d_{n} } \right|} {\sqrt {a_{n}^{2} + b_{n}^{2} + c_{n}^{2} } }}} \right. \kern-0pt} {\sqrt {a_{n}^{2} + b_{n}^{2} + c_{n}^{2} } }}$$

when $D_{i} \le g$, a point $N_{i}$ is regarded as a tunnel bottom point. Where $g$ is a variable threshold according to the designed cross-section of the tunnel shown in Fig. 6, satisfying a quadratic curve relationship. The variable threshold $g$ increases as the point approaches the centerline and decreases as it moves closer to the sidewalls. As illustrated in Fig. 7, green points represent the estimated tunnel bottom points, which the proposed method effectively removes.

#### Removal of non-tunnel structure points

**Figure 8:**
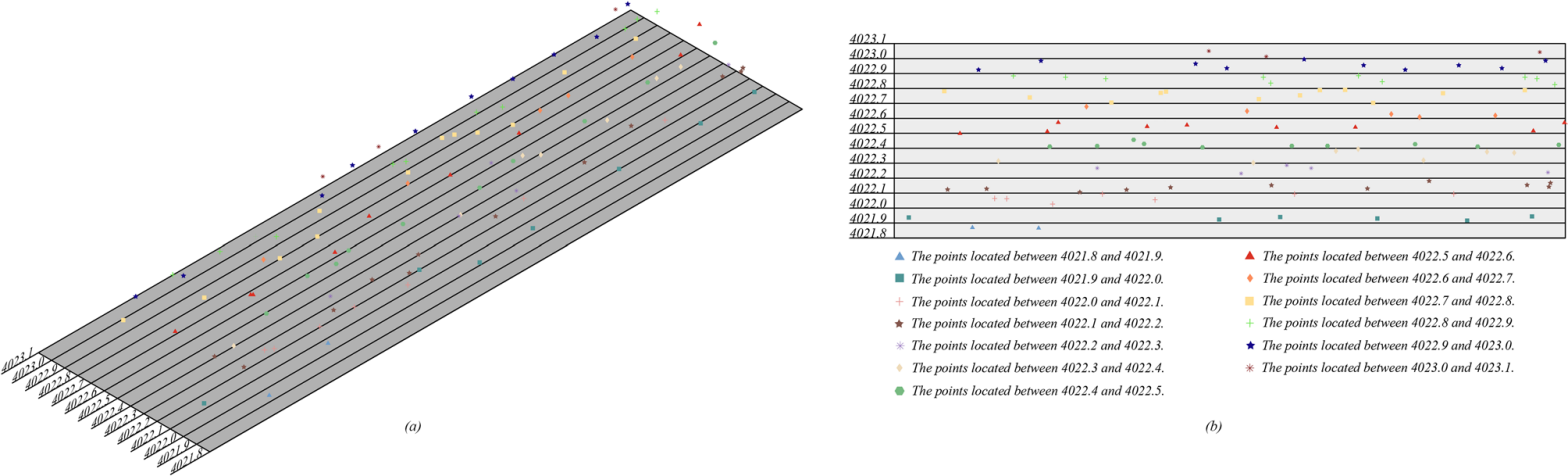
Divide unrolled point clouds into different clusters according to their $Y^{\prime\prime}$ values: (**a**) southwest isometric view; (**b**) top view.

To begin with, remove tunnel bottom points and unroll the remaining points in TSCS. The unrolled points should then be divided into various sections based on $Y^{\prime \prime }$ values displayed in Fig. 8. Different colors are used to differentiate points in different sections. All points $S_{i} (i = 1,2, \ldots ,l)$ within the same section should be rearranged into points $S_{i}^{\prime } (i = 1,2, \ldots ,l)$ according to their $X^{\prime \prime }$ values from smallest to largest. Corresponding normal vectors of points $S_{i}^{\prime } (i = 1,2, \ldots ,l)$ are represented by vectors $\vec{n}_{i}^{\prime } (i = 1,2, \ldots ,l)$.

Based on the Chinese standards TB10417-2018 (Standard for Acceptance of Tunnel Works in Railway) and JTG/T 3660-2020 (Technical Specifications for Construction of Highway Tunnel)^64,65^, it is required to excavate tunnels evenly according to their design cross-sections and ensure smoothness of primary lining surfaces. Additionally, the depth-length ratio of two protrusions should not exceed 1/20 to facilitate the installation of waterproofing boards and reinforcing mesh. The depth-length ratio of a dent to its adjacent protrusion should not exceed 1/10. Therefore, smoother lining surfaces are more conducive to point cloud denoising and hole filling. Due to the limitation of the depth-length ratio, we set an angle threshold of $3 \times \arccos ({1 \mathord{\left/ {\vphantom {1 {10}}} \right. \kern-0pt} {10}}) = 17.2175^\circ$ to remove noise points. Angles $\theta_{j} (j = 1,2, \ldots ,l - 1)$ between these normal vectors $\vec{n}_{i}^{\prime } (i = 1,2, \ldots ,l)$ can be calculated as follows:

6 $$\theta_{j} = \arccos [({{\vec{n}_{i}^{\prime } \cdot \vec{n}_{i + 1}^{\prime } )} \mathord{\left/ {\vphantom {{\vec{n}_{i}^{\prime } \cdot \vec{n}_{i + 1}^{\prime } )} {(\left| {\vec{n}_{i}^{\prime } } \right| \cdot \left| {\vec{n}_{i + 1}^{\prime } } \right|)}}} \right. \kern-0pt} {(\left| {\vec{n}_{i}^{\prime } } \right| \cdot \left| {\vec{n}_{i + 1}^{\prime } } \right|)}}]$$

when $\theta_{j} < = 17.2175^\circ$,$S_{i}^{\prime }$ and $S_{i + 1}^{\prime }$ are classified into the same cluster. When $\theta_{i,k} > 17.2175^\circ$,$S_{i}^{\prime }$ and $S_{i + 1}^{\prime }$ are classified into different clusters. Based on the changes in their normal vectors, points $S_{i}^{\prime } (i = 1,2, \ldots ,l)$ are grouped into distinct clusters.

**Figure 9:**
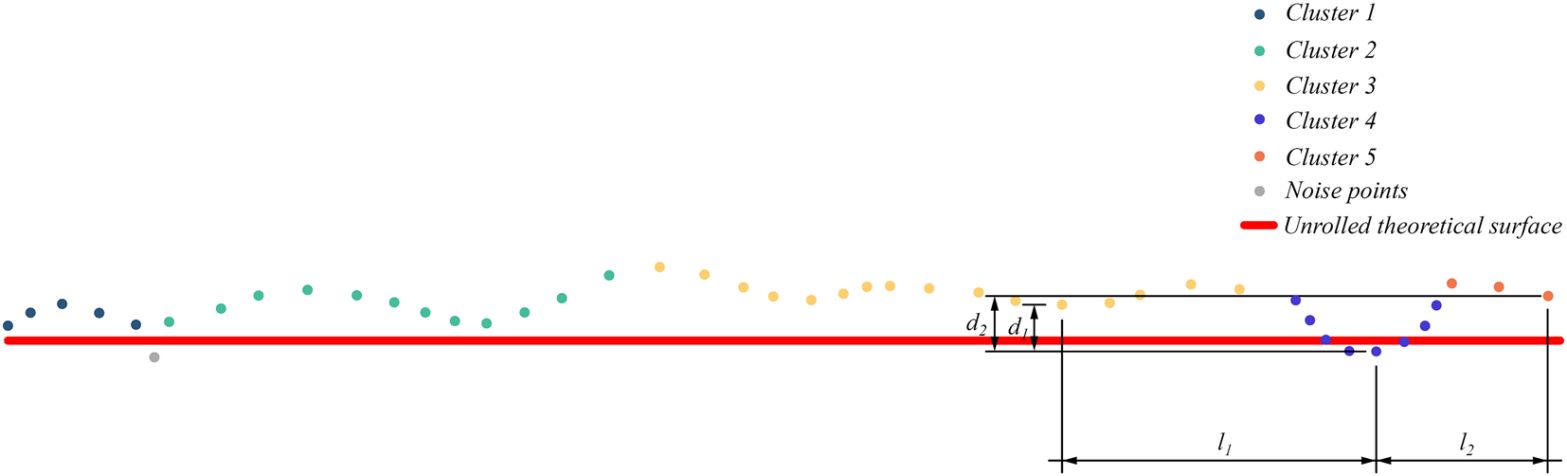
Local point cloud denoising results.

Since there is a requirement for the depth-length ratio, we further evaluate the previously segmented clusters shown in Fig. 9. The distances in the $X^{\prime \prime }$-direction from the lowest point of cluster 4 to the lowest points of two adjacent clusters 3 and 5 are denoted as $l_{1}$ and $l_{2}$, as well as the depth differences $d_{1}$ and $d_{2}$. If the following conditions are met, cluster 4 is considered noise points:

7 $${{\max (d_{1} } \mathord{\left/ {\vphantom {{\max (d_{1} } {l_{1} }}} \right. \kern-0pt} {l_{1} }},{{d_{2} } \mathord{\left/ {\vphantom {{d_{2} } {l_{2} }}} \right. \kern-0pt} {l_{2} }}) > {1 \mathord{\left/ {\vphantom {1 {20}}} \right. \kern-0pt} {20}}$$

**Figure 10:**
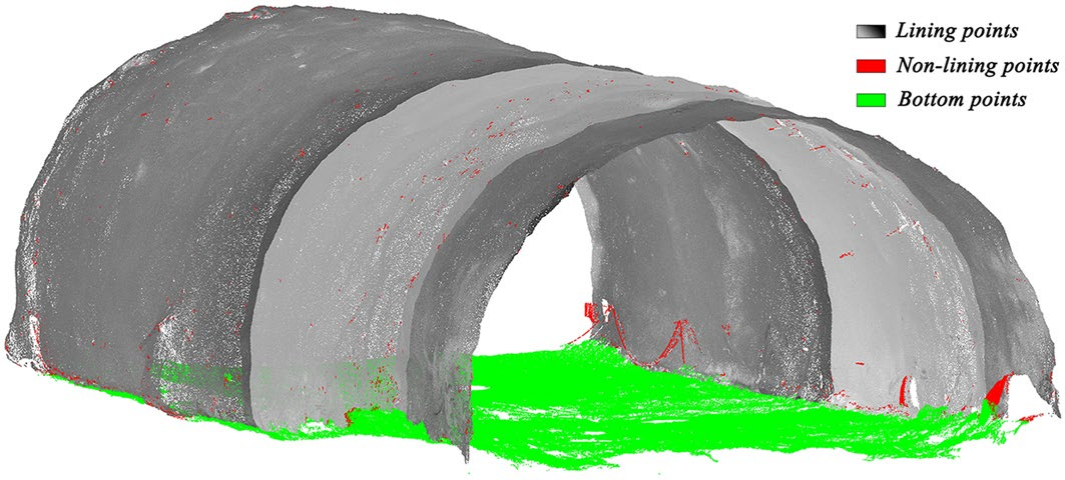
Noise reduction of the primary lining.

**Figure 11:**
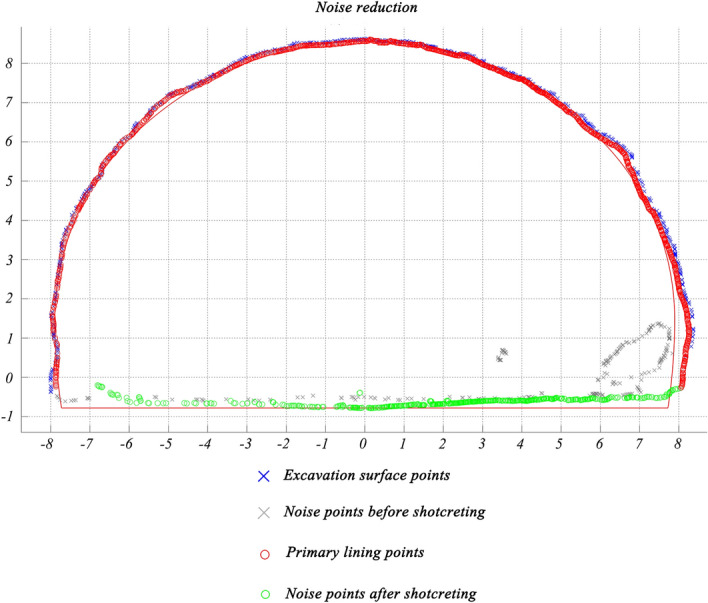
Filtering excavated rock surface and primary lining points at station DK154 + 046.14-DK154 + 046.16.

Then, the rest points are divided into different clusters according to their $X^{\prime \prime }$ values, and the estimation steps are the same as above. Finally, points after noise reduction are shown in Figs. 10 and 11. Red points represent non-tunnel structure points, while green points represent tunnel bottom points. Together, they form noise points. However, a small number of lining points close to noise points are still removed.

#### Rectangle gridding and hole filling

After noise reduction, unrolled points are divided into different grids as follows:

8 $$P_{k}^{\prime \prime } \in G_{i,j} \left\{ {\begin{array}{*{20}c} {(i - 1)\cdot w < x_{k}^{\prime \prime } < i\cdot w} \\ {(j - 1)\cdot d_{j} + {\text{initial station;}} < y_{k}^{\prime \prime } < j\cdot d_{j} + {\text{initial station}}} \\ \end{array} } \right.$$

where $P_{k}^{\prime \prime }$ is an element of the unrolled points after noise reduction; $G_{i,j}$ is an element of unrolled grids in row $i$, and column $j$.

**Figure 12:**
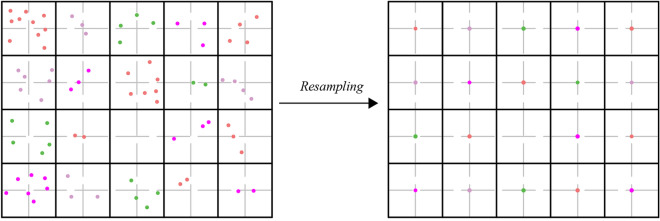
Divide point clouds into grids and resample.

As shown in Fig. 12, different colored points indicate that they belong to distinct grids. Points within a grid must be normalized to a resampled point, which represents an average $z^{\prime \prime }$ value of all points within that specific grid. Furthermore, a resampled point is projected onto the geometric centre of the corresponding expanded grid on the $X^{\prime \prime } - Y^{\prime \prime }$ plane. Resampling can reduce the impact of difficult-to-remove noise points and improve robustness.

**Figure 13:**
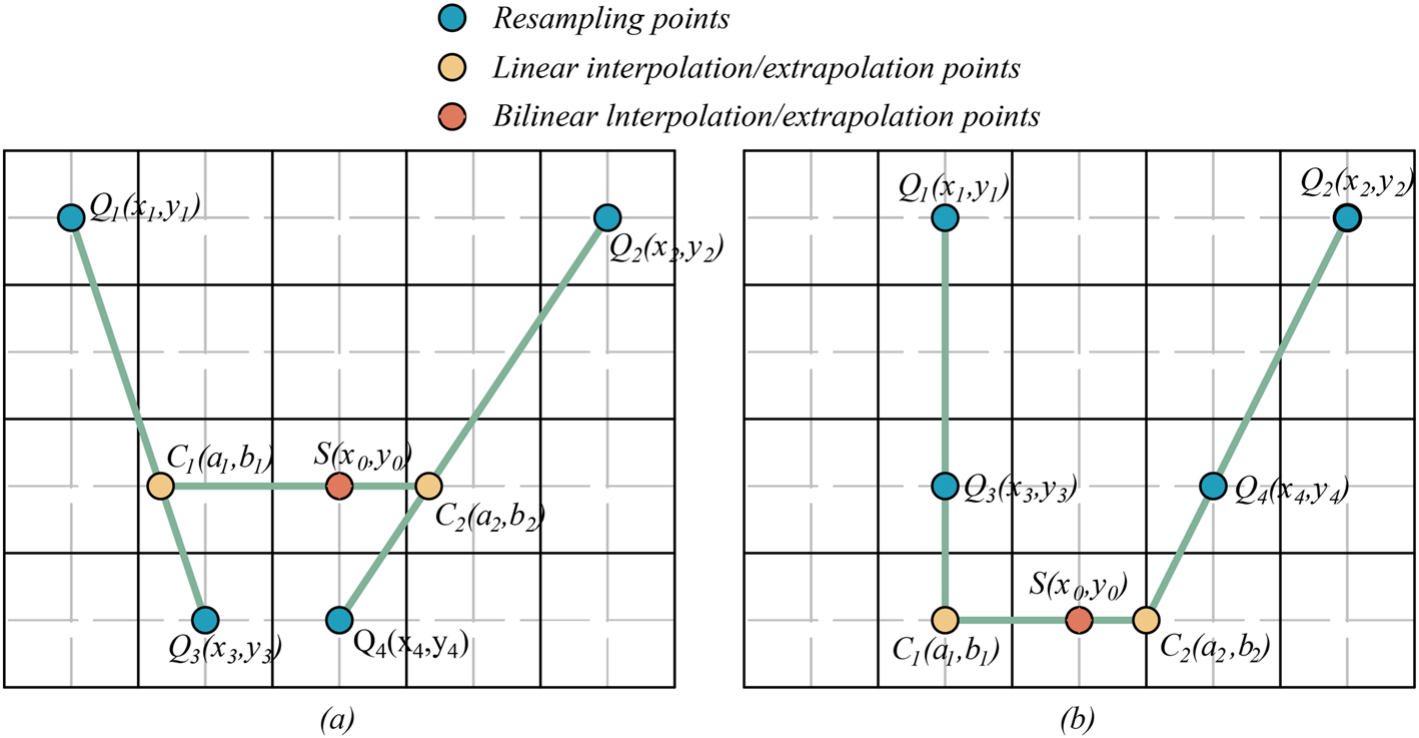
Hole filling: (**a**) gridding bilinear interpolation; (**b**) gridding bilinear extrapolation.

With holes in point clouds, there may be some grids identified as holes, where there are no resampled points available. A new bilinear interpolation/extrapolation is applied to fill these holes. Compared to the brute-force linear interpolation within raw points, this method achieves better results. In these instances, the bilinear interpolation/extrapolation point $S$ is obtained by bilinear interpolating/extrapolating the four nearest resampled points $Q_{1}$, $Q_{{2}}$, $Q_{{3}}$, and $Q_{{4}}$, as illustrated in Fig. 13(a) and (b). A gridding bilinear interpolation/extrapolation point $S$ is then utilized as the resampled point for the holes in question:

9 $$\left\{ {\begin{array}{*{20}c} {b_{1} = b_{2} = y_{0} } \\ {a_{1} = (x_{1} - x_{3} ) \times (y_{0} - y_{3} ) \times (y_{1} - y_{3} )^{ - 1} + x_{3} } \\ {a_{2} = (x_{2} - x_{4} ) \times (y_{0} - y_{4} ) \times (y_{2} - y_{4} )^{ - 1} + x_{4} } \\ \end{array} } \right.$$

10 $$\left\{ {\begin{array}{*{20}c} {f(C_{1} ) = (f(Q_{1} ) - f(Q_{3} )) \times (a_{1} - x_{3} ) \times (x_{1} - x_{3} )^{ - 1} + f(Q_{3} )} \\ {f(C_{2} ) = (f(Q_{2} ) - f(Q_{4} )) \times (a_{2} - x_{4} ) \times (x_{2} - x_{4} )^{ - 1} + f(Q_{4} )} \\ \end{array} } \right.$$

11 $$f(S) = (f(C_{1} ) - f(C_{2} )) \times (x_{0} - a_{2} ) \times (a_{1} - a_{2} )^{ - 1} + f(C_{2} )$$

among them, $(x_{i} ,y_{i} ),i = 1,2,3,4$ represent the resampled unrolled points $Q_{1}$, $Q_{{2}}$, $Q_{{3}}$, $Q_{{4}}$; $(a_{j} ,b_{j} ),j = 1, \, 2$ represent the unrolled interpolation/extrapolation points $C_{1}$,$C_{2}$; $f(C_{i} )$ represent the $z^{\prime\prime}$ value of the unrolled point $C_{i}$.

**Figure 14:**
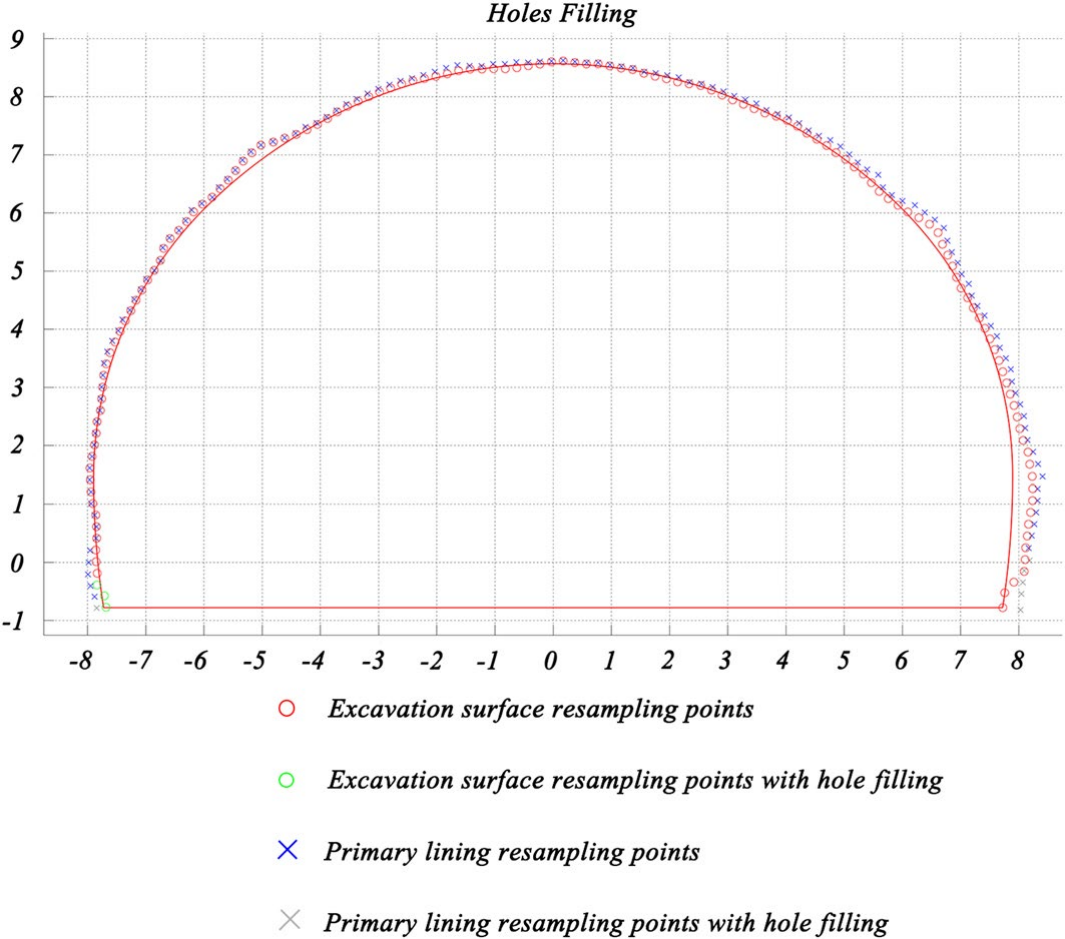
Resampled points of grids and hole filling at station DK154 + 046.

As illustrated in Fig. 14, after resampling and gridding with bilinear interpolation/extrapolation, the unrolled point should be transformed into TSCS. The result of hole filling shows that the method is of benefit to calculation.

### Establishment of a concrete consumption model

#### Computation of the volume between points and theoretical surfaces

**Figure 15:**
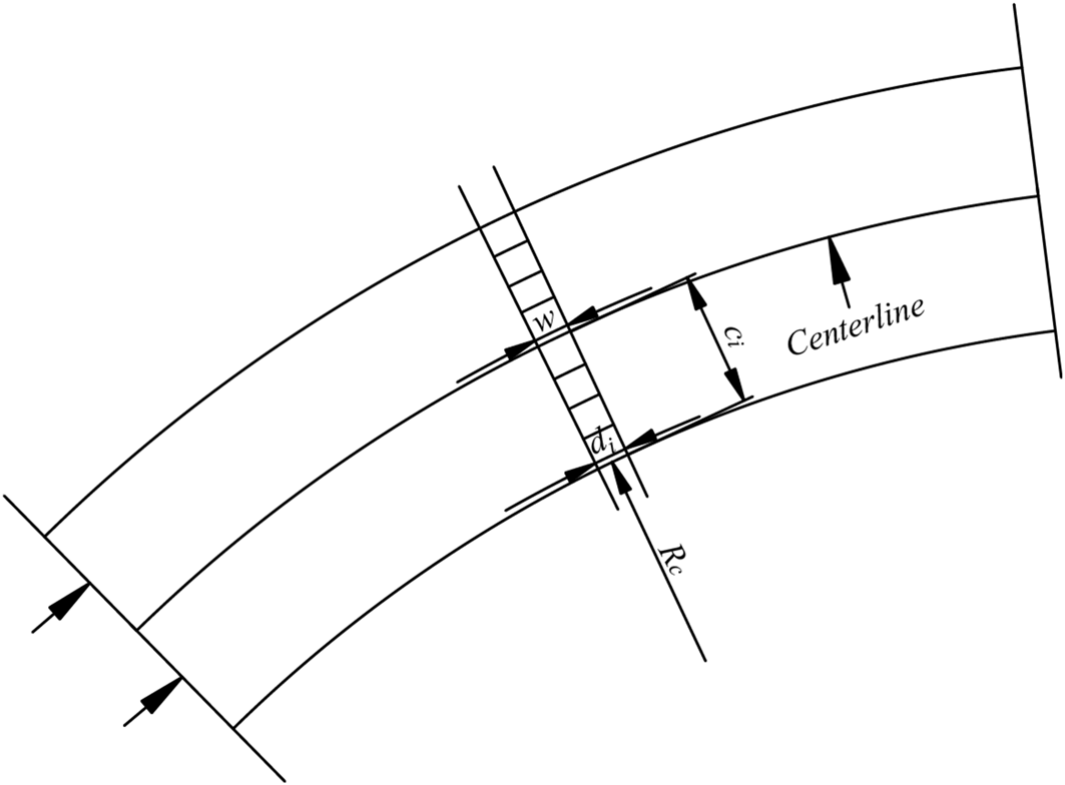
Adjusting side length of grids parallel to the centerline.

After hole filling, resampled points and grids offer new tunnel geometric information. However, it is difficult to calculate volumes directly from these resampled points and existing unrolled grids. The presence of transition and circular curves in tunnels means that the unrolled grids may not accurately reflect their areas in the geodetic coordinate system. As such, we need to correct resampled points on unrolled theoretical surfaces for increased precision. Imaginary grids exist on theoretical surfaces shown in Figs. 3a and 15. In Fig. 15, $d_{i}$ represents the grid side length parallel to the centerline, and can be calculated using the following formula:

12 $$d_{i} = {{(R_{c} + c_{i} )} \mathord{\left/ {\vphantom {{(R_{c} + c_{i} )} {R_{c} }}} \right. \kern-0pt} {R_{c} }} \cdot w$$

where $R_{c}$ is the radius of the centerline on the $X - Y$ plane; $c_{i}$ is the distance between the projection of $d_{i}$ and the centerline on the $X - Y$ plane; $w$ is the side length of this grid.

**Figure 16:**
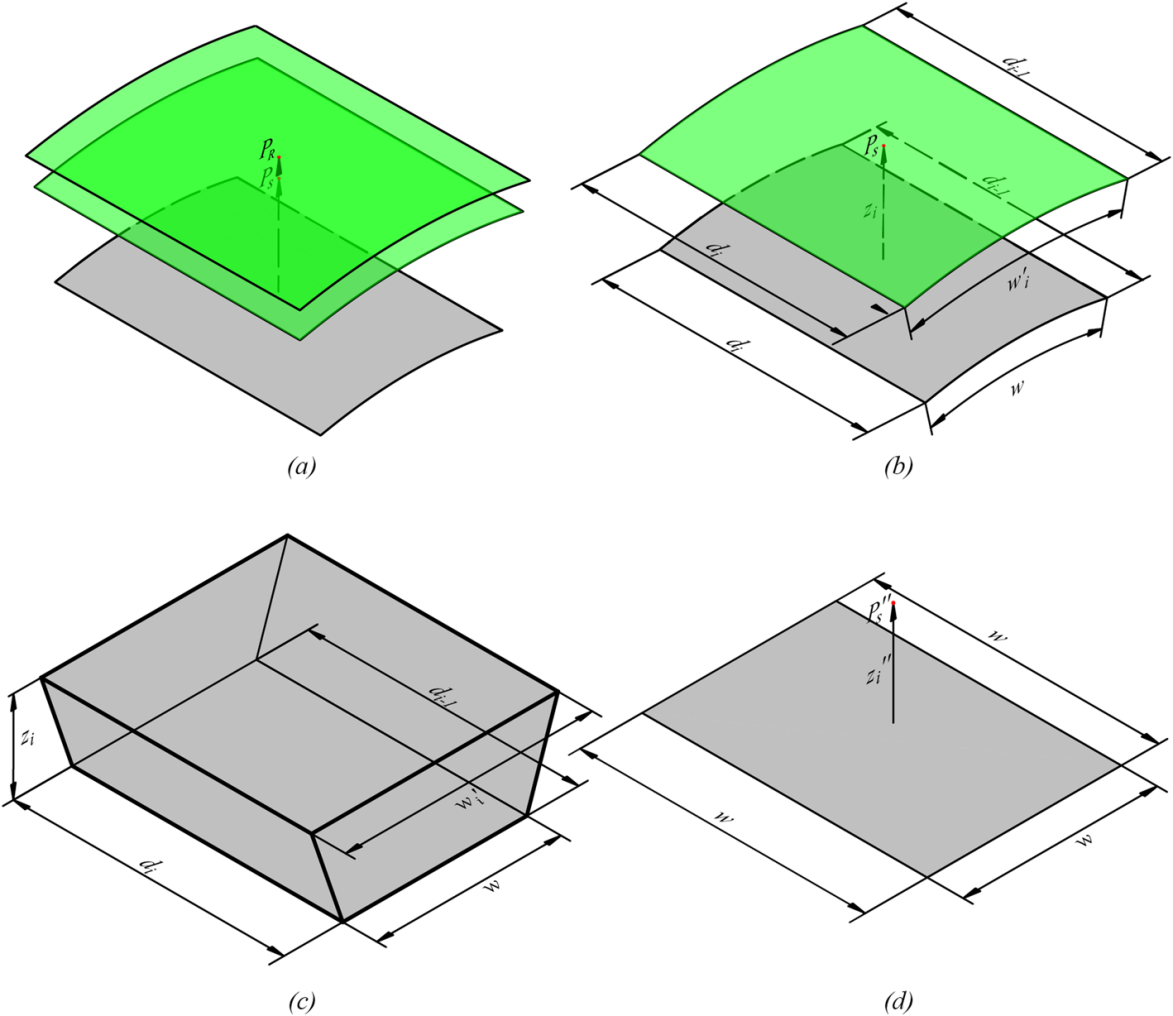
Calculation for volume: (**a**) resampled points from two scans in the geodetic coordinate system; (**b**) resampled point in the geodetic coordinate system; (**c**) expanded theoretical surface; (**d**) corrected height.

As shown in Fig. 16a,b, the gray surface is a grid on theoretical surfaces. Besides, there is a point $P_{R}$ that is a resampled excavated rock surface point of this grid and $P_{s}$ is a resampled primary lining point. $z_{i}$ is obtained by calculating the distance from point $P_{s}$ to this grid. Lengths $d_{i}$ and $d_{i - 1}$ are the side lengths in the direction of the centerline of this grid. Through the point $P_{s}$, draw the green surface which has the same cylinder axis as the gray theoretical surface in Fig. 16b. As shown in Fig. 16c, the volume between the point $P_{s}$ and the grid can be calculated as:

13 $$V_{g} = {{(w + w_{i}^{\prime } ) \cdot (d_{{i{ - }1}} + d_{i} ) \cdot z_{i} } \mathord{\left/ {\vphantom {{(w + w_{i}^{\prime } ) \cdot (d_{{i{ - }1}} + d_{i} ) \cdot z_{i} } 4}} \right. \kern-0pt} 4}$$

14 $$w_{i}^{\prime } = {{(R_{d} + z_{i} ) \cdot w} \mathord{\left/ {\vphantom {{(R_{d} + z_{i} ) \cdot w} {R_{d} }}} \right. \kern-0pt} {R_{d} }}$$

in which $V_{g}$ is the volume of a hexahedron obtained by the transformation of volume between $P_{s}$ and the grid; $z_{i}$ is the distance between $P_{s}$ and the grid; $R_{d}$ is the radius of the design cross-section; $w$, $w_{i}^{\prime }$, $d_{i}$, and $d_{i - 1}$ are separately the side lengths of the hexahedron.

As shown in Fig. 16d, the volume of the hexahedron can be represented by multiplying the corrected height $z_{i}^{\prime \prime }$ and the unrolled grid area $w^{2}$ on unrolled theoretical surfaces. The resampled point $P_{S}$ can be corrected as $P_{S}^{\prime \prime }$:

15 $$z_{i}^{\prime \prime } = {V \mathord{\left/ {\vphantom {V {w^{2} }}} \right. \kern-0pt} {w^{2} }}{ = }[{{(2R_{d} + z_{i} )(2R_{c} + c_{i} + c_{i - 1} ) \cdot z_{i} ]} \mathord{\left/ {\vphantom {{(2R_{d} + z_{i} )(2R_{c} + c_{i} + c_{i - 1} ) \cdot z_{i} ]} {(4R_{d} R_{c} )}}} \right. \kern-0pt} {(4R_{d} R_{c} )}}$$

in which, $z_{i}^{\prime \prime }$ is the corrected height used to indicate the volume.

**Figure 17:**
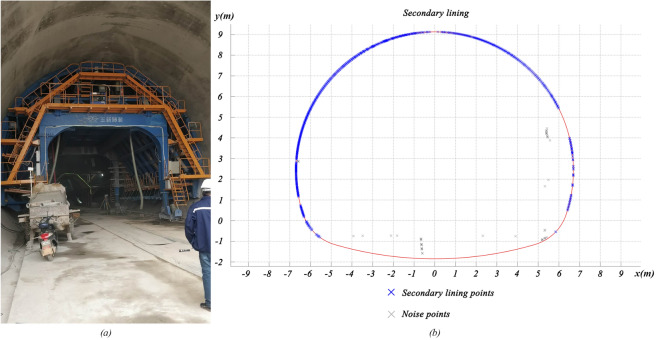
Secondary lining construction: (**a**) secondary lining trolley; (**b**) point clouds of secondary lining surfaces.

As shown in Fig. 17, the side length $w$ is a predetermined fixed value, and the volume existing between point clouds and theoretical surfaces can be computed as:

16 $$V = w^{2} \cdot \sum\limits_{i = 1}^{n} {z_{i}^{\prime \prime } }$$

#### Computation of the rebound rate of the shotcrete

Hence, the volume of shotcrete sprayed onto excavated rock surfaces can be computed in the following manner:

17 $$V_{p} = V_{be} - V_{af} - V_{sa} - V_{bm}$$

where $V_{be}$ represents the volume between excavated rock surface points and theoretical primary lining surfaces, as described in Eq. (16); $V_{af}$ represents the volume between primary lining surface points and theoretical primary lining surfaces, as also outlined in Eq. (16); $V_{sa}$ is the volume of steel arches estimated based on lengths of the steel beams used; and $V_{bm}$ is the volume of bar-mat reinforcements estimated based on lengths of the steel bars used.

We assume that shotcrete is homogeneous, and its density can be represented by $\rho$. Therefore, rebound rate can be calculated using the following formula:

18 $$R_{b} = \frac{{W_{a} - W_{p} }}{{W_{a} }} = \frac{{(V_{a} - V_{p} ) \cdot \rho }}{{V_{a} \cdot \rho }} = \frac{{V_{a} - V_{p} }}{{V_{a} }} \times 100\%$$

where $V_{a}$ and $W_{a}$ respectively represent the total volume and mass of shotcrete sprayed; $V_{p}$ and $W_{p}$ are the volume and mass of shotcrete sprayed on excavated rock surfaces.

#### Prediction of the demand for grouting concrete in the secondary lining

**Figure 18:**
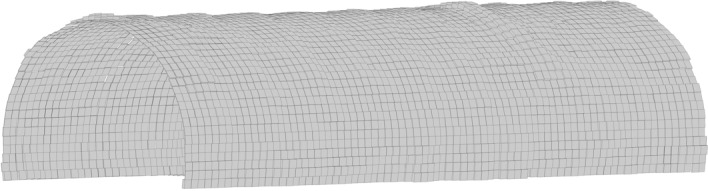
Volume calculation model of the proposed method.

Due to the use of a secondary lining trolley during the construction process, the surfaces of the secondary lining are close to the design as shown in Fig. 18. Therefore, the demand $V_{d}$ for grouting concrete in the secondary lining can be calculated with the volume $V_{af}$:

19 $$V_{d} = V_{af} - V_{wf}$$

among them, $V_{wf}$ is the estimated waterproofing material volume between the primary lining and the secondary lining.

## Experimental results and discussion

### Experimental results and comparisons

The experimental point clouds were collected from the Tiantaishan tunnel and Dafangshan tunnel with a Faro S150 laser scanner^66^. The range accuracy for TLS is ± 1 mm, and the acquisition rate is 488,000 points per second. To eliminate cumulative registration errors, two spherical prisms were used in each station scan to transfer point clouds to the geodetic coordinate system, instead of relying solely on matching and concatenation. We compare the proposed method with results obtained from the weight collection method, traditional estimation method, 3D Delaunay, and 3D Delaunay with curve fitting in terms of accuracy and efficiency. We use results obtained from the weight collection method as true values to assess the accuracy of other methods. The weight collection method involves slightly different processes and approaches during the construction of primary and secondary lining, which will be detailed in the specific experiments.

Traditional estimation method often requires a surveyor with a total station for completion^12^. A surveyor selects tunnel sections at regular intervals $\Delta z_{d}$. After selecting several sections, use a total station to collect several representative points on each tunnel section. Calculate the average linear overbreak and underbreak values of these points on each cross-section, multiplied by the designed perimeter of the tunnel cross-section, to obtain the overbreak and underbreak area for this cross-section. Then, multiply the overbreak and underbreak areas of these cross-sections by the interval $\Delta z_{d}$ and sum them to estimate the volume of the tunnel surfaces compared to the design surfaces. While this method is straightforward, it is overly coarse and challenging to achieve high accuracy.

**Figure 19:**
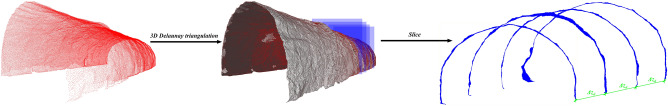
Volume calculation model of 3D Delaunay.

**Figure 20:**
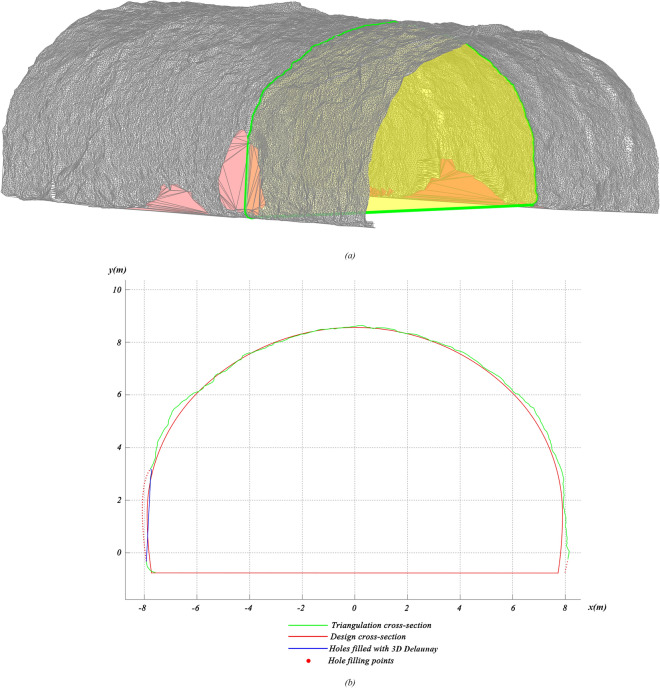
3D Delaunay triangular meshes after noise reduction: (**a**) triangular mesh model; (**b**) cross-sectional diagram.

**Figure 21:**
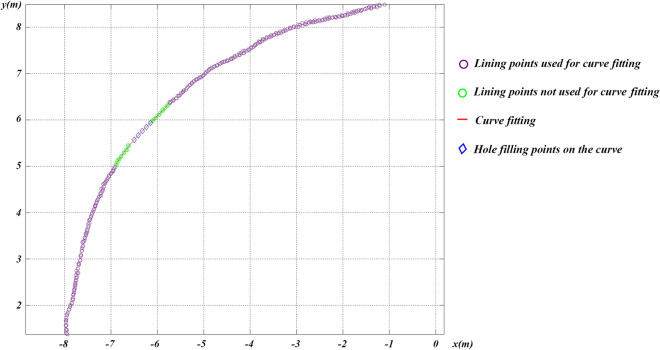
Hole filling with curve fitting.

3D Delaunay involves computing triangular meshes of point clouds before and after shotcrete application^40^, creating a polyhedral 3D model for primary linings, as shown in Fig. 19. Subsequently, slicing and integrating operations are performed on the polyhedron to determine its volume. As shown in Fig. 20a, the grey triangle meshes are generated from the point clouds, while holes with no points are generated by the surrounding points to form red triangle meshes. However, practical excavated rock surfaces and primary lining surfaces often consist of curved surfaces, which can negatively impact volume calculations. Apart from that, any digital holes will have a negative impact on volume calculations because tunnel surfaces are often not made up of continuous flat planes. As shown in Fig. 20b, hole filling is a benefit for volume calculation. As shown in Fig. 21, curve fitting is employed to fill digital holes^44^. 3D Delaunay with curve fitting exhibits higher accuracy compared to 3D Delaunay.

#### Verification of the rebound rate calculation

**Figure 22:**
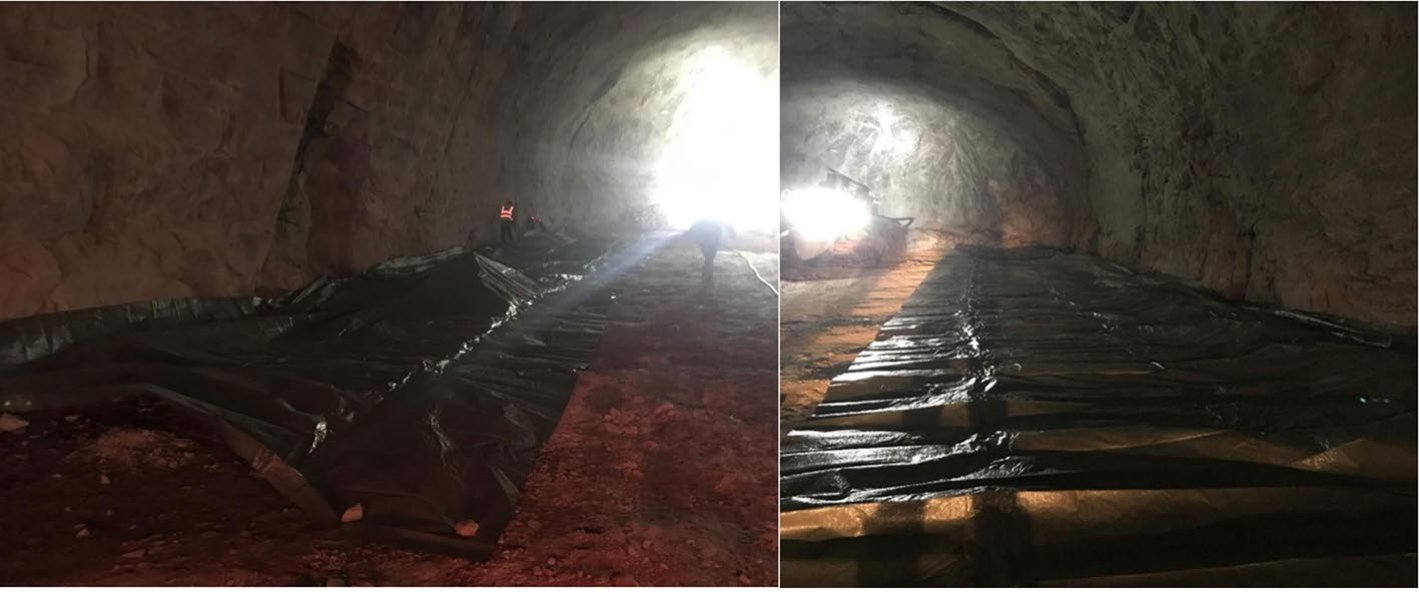
Tunnel engineering site.

The shotcrete after spraying can be divided into two types: shotcrete adheres to excavated rock surfaces and rebounds. Figure 22 illustrates the weight collection method whereby the plastic membranes were laid out on the tunnel bottom before shotcreting^48,67^. Subsequently, shotcrete was sprayed on the tunnel's arches and sidewalls. Any residual shotcrete that had fallen was then collected through the plastic membranes so that it could be weighed. The rebound rate with the weight collection method is calculated as^67^:

20 $$R_{b} = \frac{{W_{s} }}{{W_{a} }} \times 100\%$$

where $R_{b}$ is the rebound rate; $W_{s}$ is the mass of shotcrete fallen to the plastic membranes; $W_{a}$ is the total mass of shotcrete sprayed.

During primary lining construction, the weight collection method involves processes such as laying a film, collecting rebounds, and weighing. To avoid significant delays in the construction schedule, we chose to validate the method only on a selected segment measuring 38.6 m in length, rather than conducting a comprehensive comparison throughout the entire tunnel. Section YK154 + 021.8–154 + 064.0 was chosen for experimentation, where the same operator sprayed shotcrete under similar geological conditions. Additional information about these two scans can be found in Table 1. The dataset comprises a total of 264,618,903 points. Results from the weight collection method serve as true values, as documented in Table 2.

**Table 1:** Parameter settings of TLS before and after spraying shotcrete.

Stage	Station mileage (m)	Intercept mileage (m)	Intercept length (m)
Before spraying shotcrete	154 + 035.78	154 + 021.8–154 + 044.0	22.2
	154 + 053.07	154 + 044.0–154 + 055.0	11.0
	154 + 057.56	154 + 055.0–154 + 060.4	5.4
After spraying shotcrete	154 + 035.50	154 + 021.8–154 + 041.8	20.0
	154 + 043.18	154 + 041.8–154 + 055.0	13.2
	154 + 051.92	154 + 055.0–154 + 060.4	5.4

**Table 2:** The real volumes measured with the weight collection method.

Mileage	The real volume of shotcrete recorded by the flowmeter (m^3^)	The real volume of the rebounds collected by the plastic membranes (m^3^)	The real volume of the shotcrete sprayed on excavated rock surfaces (m^3^)
154 + 021.8–154 + 041.8	55.264	23.071	32.193
154 + 041.8–154 + 044.0	4.467	1.862	2.605
154 + 044.0–154 + 055.0	40.772	17.242	23.53
154 + 055.0–154 + 060.4	17.497	7.385	10.112
Total	118	49.56	68.44

**Figure 23:**
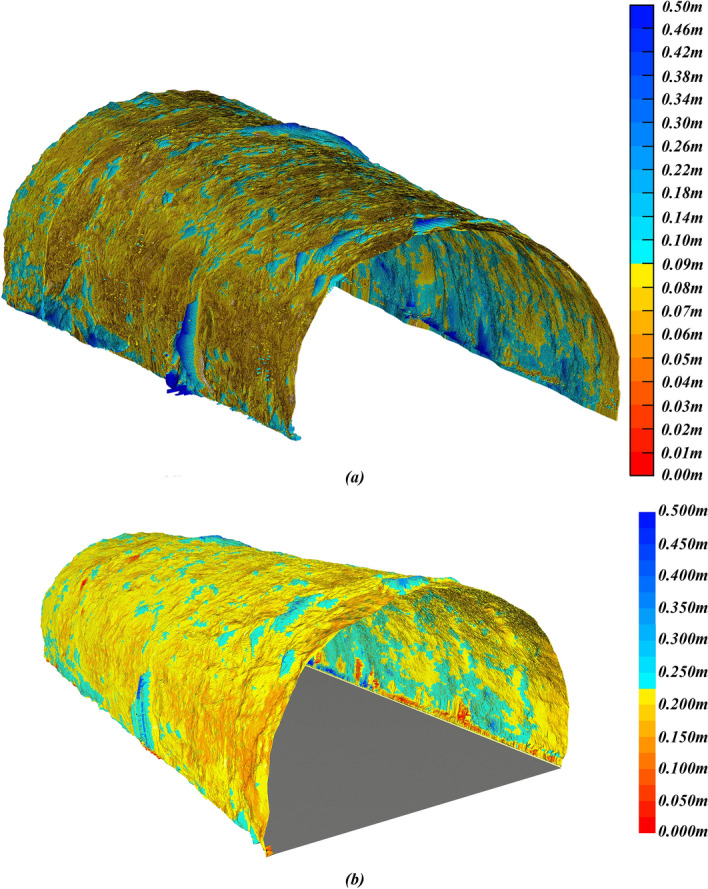
The result of the primary lining: (**a**) 3D concrete consumption model; (**b**) 3D thickness model.

As shown in Tables 3 and 4, we compare the results of multiple different methods. Calculations were performed using an i7-6700hq central processing unit (CPU). In terms of rebound rate calculation, the proposed method exhibits a relative error of 0.19%. On the other hand, 3D Delaunay yields a relative error of -10.37%. 3D Delaunay with curve fitting despite demonstrating higher accuracy with a relative error of 1.07%, still falls below the proposed method, and its computational time has reached 112.79008 seconds. The traditional estimation method exhibits a relative error of 2.02%. Additionally, due to the need to measure multiple points with a total station, this method requires more time for data collection. As a result, the proposed method outperforms 3D Delaunay with curve fitting in both speed and accuracy. Additionally, Figure 23a,b present the 3D concrete consumption model and 3d thickness model.

**Table 3:** The primary lining volumes calculated by different methods.

Mileage	The primary lining volume $V_{p}$ calculated by the proposed method (m^3^)			3D Delaunay (m^3^)	3D Delaunay with curve fitting ($\Delta z_{d} = 0.05\;{\text{m}}$) (m^3^)	True values (Weight collection method) (m^3^)
	$w = 0.05\;{\text{m}}$	$w = 0.01\;{\text{m}}$	$w = 0.005\;{\text{m}}$			
154 + 021.8–154 + 041.8	32.80206	32.61547	32.39594	28.89408	31.52246	32.193
154 + 041.8–154 + 044.0	2.63683	2.55967	2.54257	2.21478	2.47052	2.605
154 + 044.0–154 + 055.0	23.69841	23.64677	23.47908	21.96765	23.17072	23.53
154 + 055.0–154 + 060.4	10.45461	10.31900	10.24910	8.26498	10.01254	10.112
Total	69.59191	69.14091	68.66669	61.34149	67.17624	68.44

**Table 4:** The rebound rates calculated by different methods.

Method	Rebounds (m^3^)	The volume of the primary lining $V_{p}$ (m^3^)	Rebound rate $R_{b}$ (%)	Operation time (s)
True values (Weight collection method)	49.56	68.44	42.00	–
Traditional estimation with total station	46.00	72.00	38.98	About 1500
3D Delaunay	56.66	61.34	48.02	65.32813
3D Delaunay with curve fitting ($\Delta z_{d} = 0.05\;{\text{m}}$)	50.824	67.176	43.07	112.79008
Proposed method				
$w = 0.05\;{\text{m}}$	48.408	69.592	41.02	7.36890
$w = 0.01\;{\text{m}}$	48.859	69.141	41.41	20.8906
$w = 0.005\;{\text{m}}$	49.333	68.667	41.81	44.53140

With different side lengths, we have calculated the primary lining volumes at mileage 154 + 055.0–154 + 060.4 by the proposed method, as shown in Table 5. It is obvious that operation time becomes longer with the grid side length $w$ shorter. In summary, the proposed method should choose the grid side length as $w = 0.005\;{\text{m}}$. After the grid length $w < 0.005\;{\text{m}}$, the operation time of the proposed method grows significantly. Therefore, we can ensure that the accuracy is maintained at a high level and the operation time will not be too long.

**Table 5:** Errors and efficiency of the proposed method at station mileage154 + 055.0–060.4 in different grid lengths.

The side length $w$ (m)	The primary lining volume $V_{p}$ calculated by the proposed method ($m^{3}$)	True value ($m^{3}$)	The errors of the volume ($m^{3}$)	Time (s)
0.05	10.45461088	10.112	0.34261088	1.9219
0.025	10.39039891		0.27839891	2.1875
0.02	10.33651983		0.22451983	2.4063
0.01	10.31899680		0.20699680	3.5469
0.005	10.24899680		0.13699680	7.0313
0.0025	10.20597722		0.09397722	35.7188
0.002	10.17516050		0.06316050	41.3907
0.001	10.16345629		0.05145629	120.5313

#### Verification of the predicted concrete demand for the secondary lining

**Figure 24:**
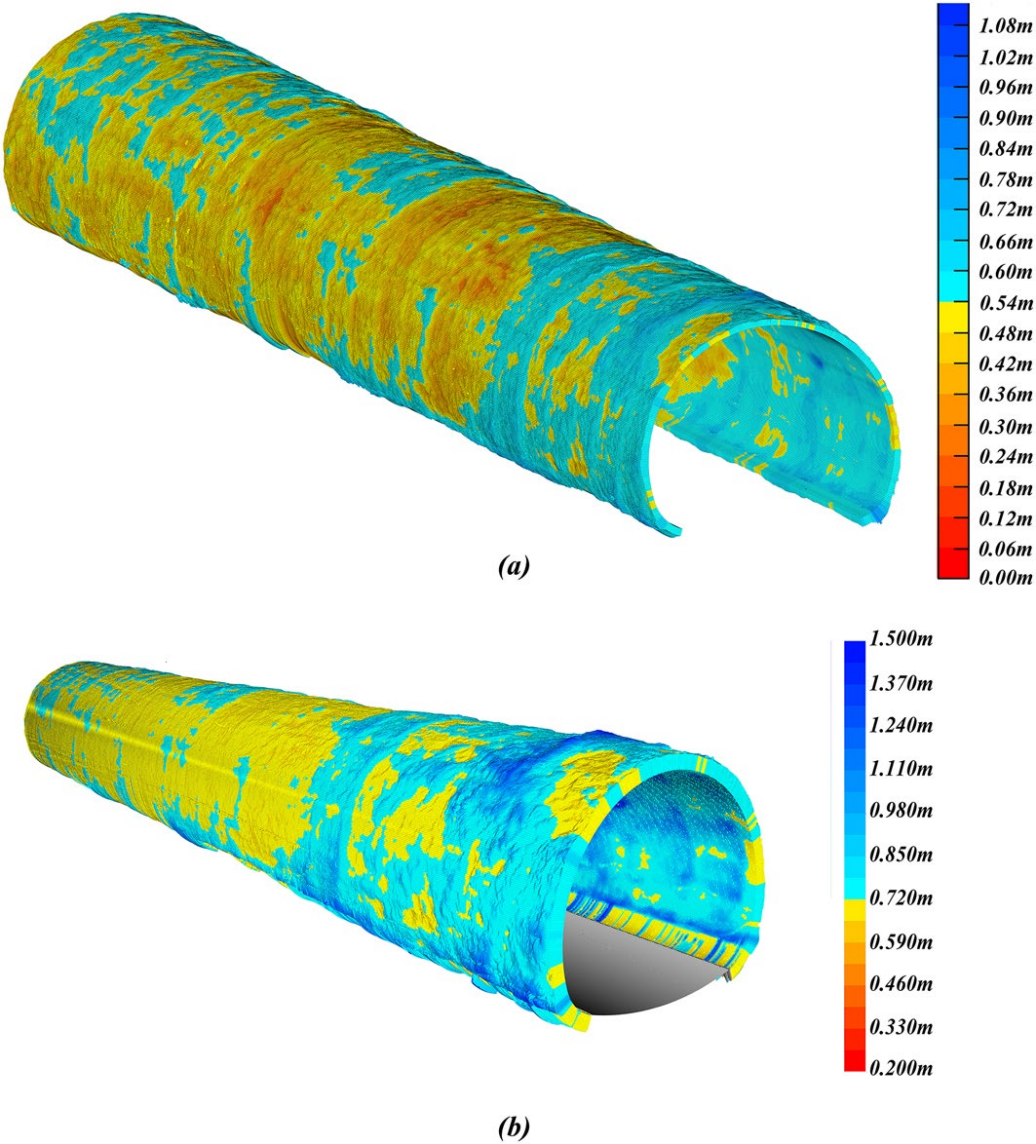
The result of the secondary lining: (**a**) 3D concrete consumption model; (**b**) 3D thickness model.

Da Fang Shan tunnel is located in Guangxi with a total length of 9647 m. The area is a typical karst topography mountainous region where karst development is intense, making it the tunnel with the highest incidence of encountering caves in the Guangxi section of the Guinan High-speed Railway. During tunnel construction, a total of 115 caves were discovered, with an average of one cave every 80 m, and the deepest cave was up to 120 m deep. We selected the DK254 + 858–DK254 + 954 section for validation, as shown in Fig. 24. The secondary lining trolley can construct 12 m in one operation, hence we use the length of each operation as the experimental length in Table 6. During secondary lining construction, the weight collection method involves weighing the trolley before and after pouring using a weighbridge, and then converting mass differences into volumes of concrete consumed during the pouring process, serving as the true values. The relative error of the proposed method in predicting the concrete demand for secondary lining is 0.15%. The relative errors of 3D Delaunay with curve fitting and traditional estimation are 2.9% and 4.0%.

**Table 6:** Demands for grouting concrete in the secondary lining with different methods.

Mileage	Proposed method ($w = 0.005m$) (m^3^)	3D Delaunay with curve fitting ($\Delta z_{d} = 0.05m$) (m^3^)	Traditional estimation with total station (m^3^)	True values (Weight collection method) (m^3^)
254 + 954 − 942	225.242	214.125	208.16	223.175
254 + 942 − 930	208.422	207.896	212.63	207.279
254 + 930 − 918	196.332	192.653	190.83	197.471
254 + 918 − 906	209.182	203.756	200.32	207.950
254 + 906 − 894	207.532	203.334	204.57	208.571
254 + 894 − 882	193.512	185.572	188.46	193.451
254 + 882 − 870	233.922	221.368	207.65	234.042
254 + 870 − 858	251.152	244.684	241.91	250.793
Total	1725.296	1673.388	1654.53	1722.732

### Discussion

As a result, the method proposed in this paper can effectively calculate the volume of irregular curved surfaces and can also have better adaptability than 3D Delaunay with curve fitting in most instances. This algorithm not only performs for multi-elliptical cross-section tunnels but also for rectangular cross-section tunnels, as straight segments on the cross-section can be considered circular curves with an infinite radius. In addition, it can also accurately predict the concrete demand for secondary lining. However, there are still some factors that may cause errors between the proposed method and true values:

1. During spraying, shotcrete may be sprayed outside the preset section. It is important to ensure that spraying is done carefully and accurately within the preset section to avoid any wastage or damage.
2. The shotcrete will be cured between spraying and scanning, and water in the shotcrete will evaporate slightly due to chemical reactions. The curing process and water evaporation during this period should be taken into consideration.
3. Plastic membranes cannot collect all rebounds, and some rebounds may fall outside them. When collecting rebounds, it is important to minimize rebounds falling outside as much as possible.
4. Slight pores inside primary linings will affect errors between the true values and the proposed method. Any pores or imperfections in the primary lining should be considered when calculating the volume of shotcrete applied to avoid significant errors or discrepancies in measurements.
5. During the construction of the secondary lining, due to the unevenness of the waterproofing material itself, there are still gaps between waterproofing materials and primary linings or between waterproofing materials and secondary linings, which leads to an overestimation in volume calculations.
6. Due to holes in the point clouds, we have performed hole filling. However, hole filling points may be different from actual surface points. While performing hole filling, it is important to keep in mind that hole filling points may differ from actual surface points, which could lead to inaccuracies in final measurements or analysis.
7. When there are too many obstructions in front of tunnel surfaces, it will reduce the point cloud density collected. Minimizing obstructions is beneficial for TLS to capture tunnel surface points. Therefore, it is necessary to move large obstructions close to the scanner before each scan.
8. The proposed method may not yield optimal results for estimating primary lining volumes in tunnels without smooth blasting. We recommend using 3D Delaunay with curve fitting when calculating the volume between excavated rock surface points and theoretical surfaces, while still employing the proposed method for other volumes. This approach enhances computational accuracy.

## Conclusions

Owing to the highly dense and accurate 3D data of TLS, it is possible to explore new ways to represent a tunnel. By scanning a tunnel under construction with TLS, an automated and novel technique is proposed to calculate concrete consumption. In this technique, point clouds of the tunnel will be expanded along the theoretical cross-section after being converted into TSCS. Noise points are removed so that they do not participate in subsequent calculations. Then the unrolled points are resampled on the expanded theoretical surfaces. In order to mitigate the adverse effects of digital holes, hole filling based on proposed gridding bilinear interpolation/extrapolation offers a more accurate result. Then, the volumes between points of two scans are estimated as the volume of the primary lining, and the rebound rate of shotcrete can be evaluated. Apart from that, we can predict the demand for concrete during the construction of secondary linings.

Experimental results show that the volume deviation between the true value and the proposed method is only 0.227 m^3^ in the 38.6 m long experimental section. Compared with the true value, the rebound rate with the proposed method has a relative deviation of only 0.19%. And the relative error in predicting the concrete demand for secondary lining is 0.15%. Therefore, the proposed method can meet the requirements of tunnel engineering. Due to the occlusion of attachments, a small quantity of non-tunnel structure points will be scanned, and digital holes in the point cloud occur at the same time. It is effective for the proposed method to reduce the inaccuracy of volume calculation caused by non-tunnel structure points and holes. c It is essential to further develop a better algorithm for noise reduction and hole filling. The proposed method needs to be applied widely in tunnel engineering for continuous improvement. These are future works planned by the authors.

## Data Availability

Data will be available by the corresponding author upon reasonable request.
